# Variability in the expression and perception of positive affect in human infancy

**DOI:** 10.1093/scan/nsad049

**Published:** 2023-09-12

**Authors:** Tobias Grossmann, Adrienne Wood

**Affiliations:** Department of Psychology, University of Virginia, Charlottesville, VA 22903, USA; Department of Psychology, University of Virginia, Charlottesville, VA 22903, USA

**Keywords:** CD38, oxytocin, positive emotion, infant temperament, brain

## Abstract

Positive emotions play a critical role in guiding human behavior and social interactions. This study examined whether and how genetic variability in the oxytocin system is linked to individual differences in expressing positive affect in human infants. Our results show that genetic variation in *CD38* (rs3796863), previously linked to increased release of oxytocin, was associated with higher rates of positive affective displays among a sample of 7-month-old infants, using established parent-report measures. Moreover, infants displaying increased levels of positive affect (smiling and laughter) also showed enhanced brain responses in the right inferior frontal cortex, a brain region previously linked to perception–action coupling, when viewing others smile at them. These findings suggest that, from early in development, genetic variation in the oxytocin system is associated with individual differences in expressed positive affect, which in turn are linked to differences in perceiving positive affect. This helps uncover the neurobiological processes accounting for variability in the expression and perception of positive affect in infancy.

## Introduction

Expressing, perceiving and sharing positive emotions plays a critical role in guiding human social behavior, interactions and relationships (Keltner and Haidt, 1999, 2001; Shiota *et al.*, 2004). The sharing of positive experiences during joint engagement is considered a key component in the evolution and development of human (pro)sociality and cooperation (Tomasello, 2020). Smiling and laughter during social interactions emerge early in human infancy and elicit positive affect and engagement in mothers and others (Sroufe and Waters, 1976; Martin and Messinger, 2018; Mazzocconi and Ginzburg, 2022). Infant smiles (and laughter) elicit approach and foster reciprocity by reinforcing (caregiver) behavior, encouraging repetition of actions and promoting social interactive exchanges (Sroufe and Waters, 1976; Martin and Messinger, 2018). Sharing positive affect during social engagement likely serves affiliative and bonding functions and has been linked to a range of positive developmental and relationship outcomes across the lifespan (Keltner and Haidt, 2001; Fredrickson *et al.*, 2004; Shiota *et al.*, 2004; Wood *et al.*, 2021).

At the neural level, when producing and perceiving smiles, adults engage brain regions such as the right inferior frontal cortex (IFC), implicated in perception–action coupling (Hennenlotter *et al.*, 2005; van der Gaag *et al.*, 2007; Bastiaansen *et al.*, 2009). Together with other behavioral and brain research on emotional resonance and mimicry in the context of smiling, this provides support for the notion that (embodied) simulation is a critical mechanism supporting positive affective engagement and mutual understanding (Keysers and Gazzola, 2007; Niedenthal *et al.*, 2010). Variability in the expression of positive affect including smiles and laughter exists among individuals and can be detected from early in human ontogeny (Sroufe and Waters, 1976). For example, using established parent-report measures, lower levels of smiling and laughter have been reported among infants who develop/are diagnosed with autism spectrum disorder (ASD) or are at a higher risk for developing ASD (Garon *et al.*, 2009; Filliter *et al.*, 2015). While findings are somewhat mixed and may depend on age and methods used to assess infant temperament [see Dawson *et al.* (2023) for a recent review], parent-reported reduced positive affect (smiling and laughter) may be considered one of the ‘early warning signs’ detectable by the end of the first year of postnatal life (Garon *et al.*, 2009; Filliter *et al.*, 2015; Dawson *et al.*, 2023). In fact, a large longitudinal study comparing infant siblings of children with ASD and typically developing infants found that parent reports of lower positive affect (and lower attention shifting) predicted a later diagnosis of autism, with this profile being stable from 6 to 24 months of age (Garon *et al.*, 2022). Children with ASD have also been shown to display dampened neural responses in the right IFC when producing and perceiving different emotional facial expressions, including smiles (Dapretto *et al.*, 2006).

However, to date, little is known about the genetic origins and neural consequences of variability in the expression of positive affect during infancy. The current study thus focuses on these questions by taking a developmental affective neuroscience approach. Specifically, we examined two main questions. First, we asked whether and how genetic variation in the oxytocin system is linked to individual differences in infants’ expression of positive affect. Second, we assessed whether individual differences in infants’ expression of positive affect are in turn linked to variability in the neural processing of positive affect in others.

Oxytocin is a neurohormone that plays a critical role in the evolution and development of affiliative social behaviors (Carter, 2014). One form of variation in the endogenous oxytocin system that has been looked at in some detail is in the ectoenzyme CD38 (Cluster of Differentiation 38). CD38 is critical to the function of endogenous oxytocin due to its regulation of calcium signaling during oxytocin release (Bartz and McInnes, 2007; Jin *et al.*, 2007; Tolomeo *et al.*, 2020). In mice, knockout models lacking the *Cd38* gene display extensive deficits in social cognition and behavior (Jin *et al.*, 2007) and have been considered a rodent model for ASD (Higashida *et al.*, 2011). In humans, naturally occurring genetic variation of the *CD38* gene in the form of the single-nucleotide polymorphism (SNP) rs3796863 has been linked to ASD, whereby the C allele of *CD38* is considered a risk allele, as it is overrepresented in ASD as compared to neurotypical populations (Lerer *et al.*, 2010; Munesue *et al.*, 2010). Neurotypical adults (parents) with the CC genotype (heightened ASD risk), as compared to non-risk A-allele carriers, display reduced plasma oxytocin and reduced caregiving sensitivity and behavior (Feldman *et al.*, 2012). Previous work with 7-month-old infants suggests that genetic variability in *CD38* interacts with breastfeeding experience (duration of exclusive breastfeeding) when differences in attention to emotional eyes are measured using eyetracking (Krol *et al.*, 2015a). Furthermore, approach and withdrawal tendencies when processing smiles and frowns vary as a function of the *CD38* genotype in 11-month-old infants using functional near-infrared spectroscopy (fNIRS) (Krol *et al.*, 2021). This suggests that genetic variability in *CD38* plays a role in the processing of social smiles. However, the more fundamental question as to whether variability in *CD38* is linked to the expression of positive affect in the infant herself has not been addressed in prior work.

The current study tested three pre-registered hypotheses in a sample of 7-month-old human infants using the Infant Behavior Questionnaire in its revised form (IBQ-R; Gartstein and Rothbart, 2003) to examine positive affect and fNIRS to measure brain responses to emotional faces. First, we hypothesized that *CD38* variants linked to greater oxytocin release in previous research (CA and AA genotypes) are associated with greater levels of positive affect, especially smiling and laughter among infants, whereas *CD38* variants linked to reduced oxytocin release and ASD in previous research (CC genotype) are associated with lower levels of positive affect (smiling and laughter). Second, we hypothesized that greater levels of smiling and laughter among infants are associated with enhanced brain responses within the right IFC when viewing other individuals’ smiles, whereas lower levels of smiling and laughter among infants are hypothesized to be associated with attenuated brain responses within the right IFC when viewing other individuals’ smiles. Third, we hypothesized that *CD38* variants linked to increased oxytocin release predict right IFC activity while viewing smiles.

This study focused on infants who were 7 months of age. The rationale for studying this age group is that, by this age, infants’ smiling and laughter have been shown to reliably occur in social contexts (Sroufe and Waters, 1976; Martin and Messinger, 2018). Moreover, social smiling and laughter increase during the second half of the first year and infants become actively involved in social activities eliciting smiles and laughter (Sroufe and Waters, 1976; Martin and Messinger, 2018).

## Material and methods

### Participants

Ninety-eight 7-month-old infants (*M*_age_= 214 days) of European descent (49 females) participated in this study. All infants resided in Leipzig, a metropolitan German city of ∼600 000 people. All infants were born at standard birth weight (>2500 grams) and gestational age (>38 weeks). No medical issues regarding development were reported at the time of testing, and there was no reported history of ASD or other neurodevelopmental disorder in any of the parents or older siblings of the infants. Parents provided written informed consent and were compensated with travel money, a toy for the infant and a printed photograph of their infant in the fNIRS cap. All procedures were approved by the Leipzig University Medical School Ethics Committee and were conducted in accordance with the Declaration of Helsinki.

### Genotyping

Saliva samples were collected from infants using assisted-collection sponges and kits (CS-2 sponges and OG-250 kits) and from mothers using passive drool collection tubes (OG-500 kit) from DNA Genotek, Ottawa, Canada. Samples were stored at room temperature until DNA isolation. DNA was isolated using the manual purification protocol from DNA Genotek. Genotyping of *CD38* rs3796863 was performed with a 5ʹ-nuclease assay using primers and probes from Applied Biosystems (TaqMan® SNP Genotyping Assay). Polymerase Chain Reaction was conducted with HotStarTaq Plus DNA Polymerase and Q-solution (Qiagen, Venlo, Netherlands) in a BioRad C1000 thermocycler with a CFX96 fluorescence reading module, using the following thermal protocol: denaturation at 95°C for 5 min; followed by cycling: 95°C for 15 s and 60°C for 1 min for 45 cycles.

#### IBQ-R.

To assess infant smiling and laughter, mothers completed the IBQ-R (Gartstein and Rothbart, 2003). The IBQ-R is a well-established and validated instrument to assess individual differences in infant temperament traits across cultures (Gartstein *et al.*, 2006, 2009). The smiling and laughter subscale assesses the extent to which an infant displays positive affect, especially smiling and laughter, during their daily activities.

#### fNIRS

Infants underwent the same fNIRS paradigm as in Grossmann *et al.* (2018). In brief, infants were presented with photographs of five Caucasian females expressing happiness, anger, fear and neutrality. These actresses were chosen from a validated and published stimulus set (FACES Collection) (Ebner *et al.*, 2010) and had expressions with average recognition accuracies ≥93.25% [see Ebner *et al.* (2010) for details]. Using Adobe Photoshop CS5 (San Jose, CA, USA), faces were placed below an oval in the center of a gray background. Baseline images consisted of photographs of five inanimate objects (vegetables) provided by Otsuka *et al.* (2007), placed centrally within the same gray background. These stimuli have been successfully used as baseline images in infant fNIRS studies concerned with face processing (Otsuka *et al.*, 2007; Nakato *et al.*, 2009, 2011; Grossmann *et al.*, 2018). The visual angles of the facial and baseline stimuli were around 15.7° × 21.7° and 16.8° × 16.8°, respectively.

The experimental fNIRS paradigm consisted of blocks of three randomized trials, each of happiness, anger and fear (Grossmann *et al.*, 2018). Each block began with an attention-getter to orient infants to the center of the screen (a video clip of a shaking rattle accompanied by sound, see Krol *et al.*, 2015b). At the beginning of each trial, a brief 150 ms bell sound (∼600 Hz) occurred to maintain infant attention. Trial presentation was pseudo-randomized such that each infant viewed every possible actress–emotion combination, no actress expressed the same emotion consecutively and no emotional expression was repeated more than twice in a row. Similar pseudo-randomization parameters were used for the baseline stimuli such that no image served as baseline twice in a row and every possible baseline–emotion combination was presented.

Baseline and face stimuli were presented in the following fashion to create dynamically changing visual stimulation: the baseline consisted of 6 s of the same photograph changing from its original size (500 ms) to a slightly larger size (∼1° increase in visual angle) (700 ms) at least five times. The face presentation consisted of 6 s of the same actress changing from a neutral expression (500 ms) to the target emotion (700 ms) five times.

Infant fNIRS data were recorded using a NIRScout system and NIRStar acquisition software (NIRx, Berlin, Germany). A custom-built elastic cap (EasyCap, Woerthsee-Etterschlag, Germany) contained 32 optodes (16 sources and 16 detectors) placed at ∼2.5 cm distance. This arrangement comprised a total of 49 channels (source–detector pairs) placed over frontal and temporal cortices of both hemispheres. Data were recorded at a sampling rate of 6.25 Hz. Near-infrared light was emitted at two wavelengths (760 and 850 nm) with a power of 20 mW/wavelength. The system automatically adjusted light intensity to provide optimal gain. Regions of interest were selected to analyze responses within the IFC in the right hemisphere. Three fNIRS channels were chosen to best capture responses from the IFC using anatomical correlations of the infant 10–20 system (Kabdebon *et al.*, 2014) and using nirsLAB software (NIRx), which estimates the projection of designated channels onto Montreal Neurological Institute space.

Infants were seated on a parent’s lap in a quiet, dimly lit room, facing a 52 × 32 cm monitor at ∼60 cm. A camera attached to the bottom of the presentation screen recorded infant behavior for online and offline tracking of attention to each trial. The fNIRS paradigm was designed and presented in Presentation software (Neurobehavioral Systems, Berkeley, CA). Videos from each session were manually coded for infant looking duration on each trial. Trials were only included if infants attended to the screen at least 4 of the 6 s for which both baseline and face stimuli were presented and were visually inspected for motion artifacts. Trials with motion artifacts were removed from further analyses. Using the MATLAB-based software Nilab2 (NIRx, Germany), data were filtered with a 0.2 Hz low-pass filter to remove fluctuations due to infant heart rate and a high-pass filter of 0.083 Hz (12 s) to remove changes too slow to be related to experimental stimuli (i.e. fluctuations due to drift). Measurements were converted into oxygenated hemoglobin (oxy-Hb) and deoxygenated hemoglobin (deoxy-Hb) absorption using the modified Beer–Lambert law. Boxcar functions corresponding to the four stimulus conditions were convolved with a standard hemodynamic response function based on the stimulus length parameter (Boynton *et al.*, 1996). The average concentration changes of oxy-Hb in response to each stimulus condition were extracted for each channel, for each individual infant.

## Results

**Fig. 1.**
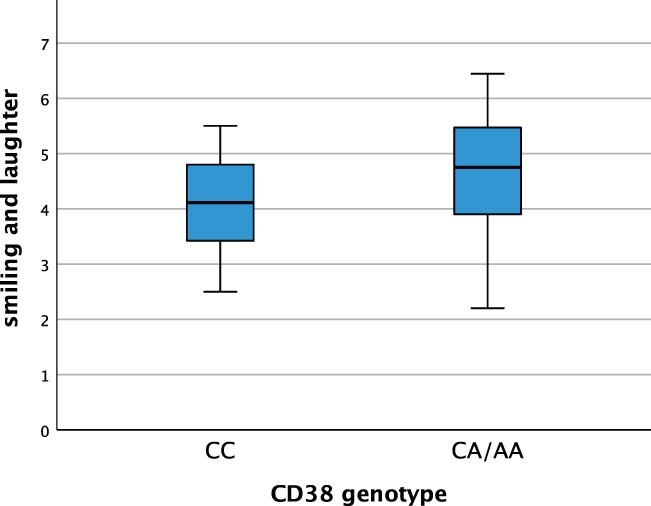
Boxplot of relation between the smiling and laughter score from the IBQ-R and CD38 genotype group (CC *vs* CA/AA).

We tested the pre-registered hypothesis (https://doi.org/10.17605/OSF.IO/3VQWT) that *CD38* variants linked to greater oxytocin release in previous research (CA and AA genotypes) are associated with greater levels of positive affect (assessed through maternal report), especially smiling and laughter among infants, whereas *CD38* variants linked to reduced oxytocin release and ASD in previous research (CC genotype) are associated with lower levels of positive affect (smiling and laughter). Specifically, we conducted a pre-registered one-way Analysis of Variance (ANOVA) with the smiling and laughter score from the IBQ-R as the within-subjects factor and the CD genotype (CC *vs* CA/AA) as the between-subjects factor. From the full sample of 98 7-month-old infants, 83 infants were included in this analysis because they provided the IBQ-R data required for this analysis (*n* = 15 failed to provide IBQ-R data and are therefore not included). In line with the pre-registered hypothesis, our analysis revealed a significant effect of genotype, *F*(1, 82) = 7.23, *P* = 0.009, η*p*^2^ = 0.082, whereby infants with the CA and AA genotypes indeed displayed higher levels of smiling and laughter (*n =* 48; *M =* 4.67; SE* = *0.14) than infants with the CC genotype (*n =* 35; *M =* 4.10; SE* =* 0.15) (see Figure 1).

**Fig. 2.**
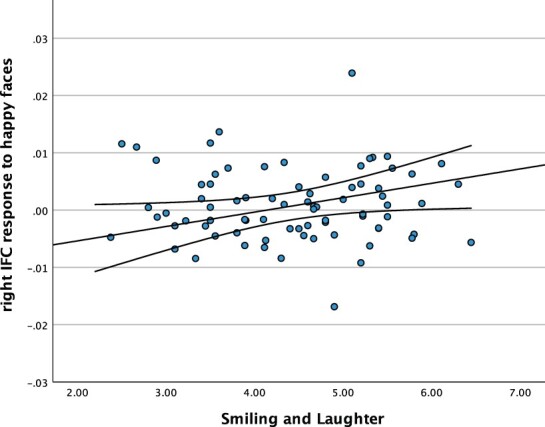
Scatterplot of relation between the smiling and laughter score from the IBQ-R and infants’ response in the right IFC in micromolar.

We further tested the pre-registered hypothesis that greater levels of smiling and laughter among infants are associated with enhanced brain responses within the right IFC when viewing other individuals’ smiles. From the full sample of 98 7-month-old infants, 79 infants were included in this analysis because their fNIRS data fulfilled our pre-defined inclusion criteria (*n* = 19 failed to provide sufficient, artifact-free fNIRS data and are therefore not included). We used a pre-registered stepwise multiple linear regression with the smiling and laughter score from the IBQ-R as the regressor variable and brain responses to happy, angry and fearful faces in the IFC within the right hemisphere as predictor variables. In line with this hypothesis, this analysis revealed that greater levels of smiling and laughter among infants are indeed associated with enhanced brain responses within the right IFC when viewing smiles, *R* = 0.229; *F*(1, 79) = 4.302; *P* = 0.041 (see Figure 2). As expected, brain responses to angry and fearful faces were not predictive of IBQ-R smiling and laughter scores, suggesting that it is specifically the experience of producing frequent positive displays that predict neural responses to perceiving positive displays.

In an additional pre-registered analysis, we examined whether the *CD38* genotype has a direct effect on the IFC response to happy faces in the right hemisphere by conducting a one-way ANOVA with IFC responses in the right hemisphere as the dependent variable and the CD genotype (CC *vs* CA/AA) as the between-subjects factor. This analysis did not reveal a significant effect of genotype, *P* = 0.75. Thus, although the *CD38* genotype predicts smiling and laughter behavior, and smiling and laughter behavior predicts neural response to smiles, there is no evidence for a direct association between genotype and neural activity.

Further analysis revealed that maternal *CD38* variation was associated neither with their infants’ right IFC response to smiling faces (*P* = 0.252) nor with their infants’ displays of positive affect (*P* = 0.512), indicating that associations effects were specific to infant *CD38* variation.

Finally, we conducted a multiple regression using both infant *CD38* genotype and brain response in the right IFC to smiling as predictors of infant positive affect (smiling and laughter). This analysis revealed that infant *CD38* genotype and brain response in the right IFC to smiling significantly predicted infant positive affect, *R* = 0.377; *F*(2, 79) = 6.391; *P* = 0.003 (full model). Furthermore, this analysis showed that both infant *CD38* genotype, β = 0.300, *t*(79) = 2.844, *P* = 0.003, and the brain response in the right IFC to smiling, β = 0.22, *t*(79) = 2.169, *P* = 0.033, were significant predictors of positive affect.

## Discussion

The current study investigated the genetic origins and the neural consequences of variability in the expression of positive affect in 7-month-old human infants by employing a developmental affective neuroscience approach. Our results confirm two of our pre-registered hypotheses. First, we show that *CD38* variants linked to greater oxytocin release (CA and AA genotypes) were indeed associated with greater levels of smiling and laughter among infants, whereas the *CD38* variant linked to reduced oxytocin release and ASD (CC genotype) was associated with lower levels of smiling and laughter. Second, we show that greater levels of smiling and laughter among infants were associated with enhanced brain responses within the right IFC when viewing smiles, whereas lower levels of smiling and laughter among infants were associated with attenuated brain responses within the right IFC when viewing smiles. These findings demonstrate that, from early in human development, genetic variation in the oxytocin system is associated with expressed positive affect, which in turn links to individual differences in perceiving positive affect.

Considering that expressing and sharing positive emotions play a critical role in guiding human social interactions and relationships across development (Keltner and Haidt, 1999, 2001; Shiota *et al.*, 2004), identifying factors linked to variability in the expression and perception of positive affect in infancy provides important neurodevelopmental insights into the nature of positive affect. Our findings show that diminished positive affect among human infants is linked to a genetic variant (CC genotype) in the oxytocin system previously linked to autism (Lerer *et al.*, 2010; Munesue *et al.*, 2010). This result is in line with prior work showing that lower levels of smiling and laughter are seen among infants who develop/are diagnosed with ASD or are at a higher risk for developing ASD (Garon *et al.*, 2009; Filliter *et al.*, 2015). Indeed, reduced positive affect is considered one of the ‘early warning signs’ detectable in the first year of postnatal life. In this context, it is important to mention that the current study relied on a group of neurotypical infants without a prior history of autism or other neurodevelopmental disorders in their families.

At the neural level, our results show that infants who are reported to smile and laugh more show enhanced brain responses within the right IFC when viewing others smile at them. This agrees with previous studies showing that adults engage the right IFC, implicated in perception–action coupling, when producing and perceiving smiles (Hennenlotter *et al.*, 2005; van der Gaag *et al.*, 2007; Bastiaansen *et al.*, 2009). The current neural-level findings with infants also converge with research showing that children with autism display dampened neural responses in the right IFC when producing and perceiving different emotional facial expressions, including smiles (Dapretto *et al.*, 2006).

Thus, together with other behavioral and brain research on emotional resonance and mimicry in the context of smiling (Keysers and Gazzola, 2007; Niedenthal *et al.*, 2010), our findings provide support for the notion that perception–action coupling might be a critical mechanism, supporting positive affective engagement from early in human ontogeny. It may also be the case that facial perception–action coupling emerges through experience with adults visibly imitating facial displays produced by the infant (de Klerk *et al.*, 2019). Infants who infrequently smile and laugh experience fewer opportunities to form those perception–action associations and may therefore show reduced neural sensorimotor simulation while viewing faces. Future longitudinal work should unravel the causal relationship between smile and laughter production and neural simulation.

The limitations of the present study point to several future directions. We cannot rule out the possibility that parents who carry the oxytocin-increasing *CD38* variants interact with their infants differently; thus, the effect of the gene on smiling and laughter behavior may be due to variations in family environment (Planalp *et al.*, 2017). However, we were able to show that maternal *CD38* variation was associated neither with their infants’ right IFC response to smiling faces nor with their infants’ displays of positive affect, suggesting that the effects were specific to infant *CD38* variation. Future work should use behavioral observation, rather than parent (maternal) reports as used in the current study, to see whether *CD38* variants predict differences in parent behavior, infant behavior or both. Future work should also compare infant neural responses to rewarding social and non-social stimuli to determine whether infant smiling and laughter behavior are associated with responses to perceived smiles, specifically, or rewarding stimuli, generally.

Smiles and laughter are important tools young preverbal infants possess for eliciting and rewarding caregivers’ positive engagement (Mazzocconi and Ginzburg, 2022). Understanding the genetic moderators of these behaviors—and, indirectly, the neurohormonal mechanisms by which genes modulate behavior—may thus inform interventions for infants with a heightened risk for autism who display reduced levels of smiling and laughter. This work also highlights the possible long-term consequences that reduced positive affect may have on social interactions. Together, the current findings contribute to our understanding of the neurobiological processes that account for variability in the expression and perception of positive affect during infancy.

## Data Availability

The data underlying this article will be shared on reasonable request to the corresponding author.
